# Association between adult education, brain volume and dementia risk: longitudinal cohort study of UK Biobank participants

**DOI:** 10.1007/s11357-024-01285-y

**Published:** 2024-07-19

**Authors:** Jiayin Jin, Andrew Sommerlad, Naaheed Mukadam

**Affiliations:** 1 Queen Square Institute of Neurology, University College London, London, UK; 2 Division of Psychiatry, University College London, London, UK; 3 Camden and Islington NHS Foundation Trust, London, UK

**Keywords:** Dementia, Education, Risk, Cohort

## Abstract

Less childhood education is a potentially modifiable risk factor for developing incident dementia but it is not known if education in later life is protective. We sought to add to previous work by testing the association between adult education and brain volume as well as exploring the impact of continuing adult education versus intermittent participation. We used data from participants of the UK Biobank cohort, with no prevalent dementia who were asked about adult education participation at baseline and at follow-up. Dementia status was ascertained from self-report or electronic health records. Cox proportional hazards models were built to estimate hazard ratios (HRs) between participation in adult education and dementia risk. In 499,337 participants aged between 40 and 69 at baseline with 13.2 years mean follow-up, in analyses adjusted for age, sex, education, deprivation, ethnicity, hypertension, diabetes, ethnicity, obesity, smoking, alcohol use, physical inactivity and social isolation, we replicated previous findings of a protective effect of adult education on dementia risk (HR 0.82, 95% CI 0.74–0.90, *P* < 0.001), and showed a trend towards protection against dementia if adult education was continued rather than intermittent. Additionally, adult education did not impact on total brain volume (coefficient − 657.4, 95% CI − 2795.1 to 1480.3, *P* = 0.547) but it was associated with increased hippocampal volume (coefficient 33.9, 95% CI 8.9 to 59.0, *P* = 0.008) indicating a potential mechanism for protection against dementia. We have added evidence indicating that continuing adult education participation may be beneficial, although numbers for this analysis were very small. Analysis of brain volume indicated that adult education may have a protective effect by preserving hippocampal size or slowing volume loss, in line with the cognitive reserve hypothesis.

**Supplementary Information:**

The online version contains supplementary material available at 10.1007/s11357-024-01285-y.

## Introduction

There are currently over 55 million people with dementia worldwide, most of whom are from low- and middle-income countries [1], with consequences for those developing dementia and to their family carers and social communities. Identifying preventative strategies to reduce dementia incidence is a public health priority. There is consistent evidence that low educational attainment in childhood is associated with increased risk of dementia [2]. Studies have suggested that the higher levels of education received during childhood have translated to lower dementia incidence in successive cohorts of older adults [3]. However, it is unclear to what extent education during adulthood after completion of school, college and/or university studies, hereby referred to as adult education, is associated with dementia risk.

One UK study with 10 years of follow-up found that participation in cognitive or social activities such as adult education, art or music class was associated with lower risk of dementia [4]. A further recent longitudinal prospective cohort study reported that among a total of 19 leisure activities measurements, participation in adult literacy and mental activities were both associated with around 10% lower incident dementia risk [5]. Furthermore, a systematic review on cognitive activities such as playing chess found increasing evidence of the positive impact of these activities on reducing dementia risk and cognitive impairment in later life [6].

However, studies included in this review had limited follow-up duration; five of the seven studies had less than 6 years interval between activity measurement and dementia assessment and the two studies with 9 and 12 years of follow-up found null results [7]. This suggests that some previous studies with short follow-up may have been influenced by reverse causation bias whereby low participation in adult education or other activities is a consequence of the prodromal dementia phase, rather than being causally associated with dementia [7]; therefore, studies with longer follow-up are needed to elucidate causal relationships.

A previous study using UK Biobank data found a protective effect of adult education on dementia risk and some association with cognitive test scores [8]. The cognitive reserve hypothesis posits that education provides the brain with the ability to accumulate neuropathology without demonstrating the phenotype of cognitive decline [9]. Childhood education has been found to be associated with both total brain volume [10] and hippocampal volume [11] in older adults. In this study, we therefore aimed to add to previous work by testing the association between adult education and brain volume as well as exploring the impact of continuing adult education versus intermittent participation. We investigated whether there is an association between adult education and incident dementia after 13 years of follow-up, and then assessed whether changes in adult education such as stopping participation was differentially associated with dementia risk.

## Methods

### Participant sample

This study used secondary data from the UK Biobank (UKB) which is a cohort of over half a million participants who were recruited throughout England, Wales and Scotland. All participants were registered at an assessment centre from 2006–2010, aged between 40 and 69 years old [12]. Participants completed online questionnaires and interviews about their demographic and health conditions.

Furthermore, UK Biobank linked participants’ existing health records from general practice and hospitals and mortality records. UK Biobank received approval from the National Information Governance Board for Health and Social Care and North West—Haydock Research Ethics Committee with the reference number 21/NW/0157. All participants gave informed consent electronically at baseline assessment. We applied for use of the data through application number 40055 and were granted permission after internal review by submitting the Material Transfer Agreement form for data and samples. We restricted our sample to all participants who did not have a diagnosis of dementia at baseline.

### Adult education

Participation in adult education class was the main exposure variable (UKB variable n_6160) and assessment was conducted at baseline between 2006 and 2010 and at a follow-up visit conducted between 2014 and 2019. At each of these assessments, participants were asked to answer the question “which of the following do you attend once a week or more often?” and provided with five options, one of which was “adult education classes”. We classified participants as participating in adult education at baseline if they had selected this as one of the leisure activities they participated in at baseline assessment, regardless of participation in other categories of activity. Those who did not select adult education classes were categorised as not participating.

To explore whether starting or stopping participation in adult education class, or continued participation, was associated with dementia risk, we used data from the follow-up assessment. Participants were categorised as (1) not participating in adult education at either time point, (2) participating in adult education only at baseline and (3) participating only at the follow-up assessment or had persistent participation from baseline assessment to the second follow-up.

### Dementia status

We ascertained incidence of all-cause dementia as defined by ICD-10 codes F00 (dementia in Alzheimer’s disease), F01 (vascular dementia), F02 (dementia in other diseases) and F03 (unspecified dementia) [13] by self-report or from three linked electronic health records (UKB variables n_130837, n_130839, n_130841, n_130843, n_131037). The electronic health records were NHS Digital’s Hospital Episode Statistics (HES) which include clinical diagnoses entered during routine clinical contact in inpatient and outpatient NHS settings [14]; primary care and mortality records [15] using defined ICD 9 and ICD 10 codes [16]. HES has 78% sensitivity and 92% specificity for dementia diagnosis [17]. The accuracy of dementia diagnoses in UK Biobank was also demonstrated by high positive predictive value estimates of 80–87% when compared against clinician-rated assessment of clinical notes [13].

### Brain volume

Over 40,000 UK Biobank participants have had three structural brain MRI scans: T1, T2 fluid attenuation inversion recovery (FLAIR) and susceptibility-weighted MRI (swMRI). T1 scans allow precise volumetric measures of the whole brain, as well as specific cortical and subcortical regions. An automated processing pipeline for brain image analysis and quality control was established for UKB at the University of Oxford’s Wellcome Centre for Integrative Neuroimaging (WIN/FMRIB) [18]. This pipeline is primarily based around FSL (FMRIB’s Software Library), and other packages such as FreeSurfer [19]. We used total brain volume (normalised for head size, variable 25009) and hippocampal volume in mm^3^ (combining volumes for left and right structures) (UKB variables 26562 and 26593) as calculated from T1 images carried out at the follow-up visit, using Automatic Segmentation (ASEG) [20], as two of our secondary outcome measures, adjusting for all covariates.

### Covariates

Demographic information (age, sex, Townsend scores, education, ethnicity), health conditions (diabetes, hypertension, obesity), health behaviours (current smoking status, excess alcohol consumption, physical inactivity) and social isolation were considered covariates (classification of covariates is detailed in Table 1). They were included as they are all associated with risk of dementia [21]. Age and sex were taken from baseline for all models and other covariates were taken from the time of exposure, either baseline or follow-up.

**Table 1:** How each covariate was derived [21]

Covariates	Measurement in UK Biobank	Use in analysis
Townsend score	Measured material deprivation (unemployment, overcrowding, non-car ownership and non-home ownership) within participants. Positive value indicates high material deprivation, negative value indicates affluence [35]	The score as provided by UK Biobank was divided into 5 quintiles and used to adjust models
Hypertension	Self-reported diagnosis or under anti-hypertensive medication treatment	Self-report or currently using anti-hypertensive medications was considered having hypertension [36]
Diabetes	Self-report or use of diabetes medication	Either self-report or currently using medications was considered having diabetes
Obesity	Height and weight were measured at baseline	BMI(Body Mass Index) ≥ 30 = obesity [37]
Education	Self-reported age of finishing full-time education and highest qualification	Participants were categorised as being educated up to 16 years old or having education over 16 years old
Ethnicity	Self-identified ethnicity	Categorised into White, South-Asian, Black and other ethnic group
Excess alcohol consumption	Self-report drinking frequency, quantity and type of drinks consumed per week [38]	Total units of alcohol consumed per week were calculated by quantity*frequency of self-reported weekly drinking. Participants were categorised as having excess alcohol intake (> 21 units per week) or drinking within moderate limits (≤ 21 units per week)
Current smoking status	Self-report as current, previous or never smokers	Participants were categorised as current smokers or non-current smokers [39]
Physical inactivity	Self-reported physical activity duration, intensity and frequency over the past four weeks duration	Physical activity was defined by calculating the weighted MET-minutes per week [40]. Participant were categorised as meeting the WHO’s guidance for physical activity or not meeting the guidance [41]
Social isolation	Self-reported frequency of contact with friends and families and self-reported living situation (alone or living with others) [42]	Participants were categorised as having daily/almost daily social contact and those with less frequent social contact

### Statistical analysis

We first described the demographic characteristics of participants based on their participation status in adult education class and the frequencies of participation consistency.

To calculate follow-up times from baseline, the start date was the date participants first attended the assessment centre. The end date was the earliest of date of all-cause dementia diagnosed, date lost to follow-up, date of death and the last date of available electronic health record data (22 September 2022). Similarly, to calculate follow-up times from the follow-up visit, we excluded participants who had dementia before or at the date of the second follow-up. The start date was the date participants attended the assessment centre in the second follow-up. The end date remained the same. For all models, the *P* values were set with a statistical significance of less than 0.05. The analyses were all performed using STATA version 17.0.

#### Analysis 1: association of adult education at baseline with dementia incidence

A Cox regression analysis was conducted using participation in adult education at baseline as the main exposure variable and all-cause dementia as the main outcome variable. Results were presented as hazard ratios (HRs) for dementia incidence. Model 1 was unadjusted; model 2 was adjusted for age and sex; model 3 was additionally adjusted for education, Townsend score and ethnicity; model 4 was additionally adjusted for hypertension, diabetes, obesity, alcohol, smoking and physical inactivity; model 5 was also adjusted for social isolation.

#### Analysis 2: association of persistent adult education participation with dementia incidence

To investigate whether persistent participation in adult education is associated with dementia risk, another Cox regression analysis was run using participation status during both baseline and the follow-up as the main exposure variable and all cause dementia diagnosis as the main outcome variable. Non-participation in adult education at both time points was used as the reference category. Participation in adult education only at baseline was used as the first category, participation in adult education only at follow-up was used as the second category and persistent participation at both baseline and follow-up was used as the third category. Sequential adjustment of models was as detailed above.

#### Analysis 3: association of adult education participation with brain volume

To investigate the association between adult education and brain volume, we summarised mean brain volume and hippocampal volume at follow-up in those who participated in and did not participate in adult education at baseline. We then conducted linear regression analyses using adult education as the exposure and total brain volume as the outcome, adjusting for age, sex and all other covariates used in previous analyses. We repeated the same analyses using total hippocampal volume.

#### Sensitivity analysis

We only excluded people with dementia at baseline in our primary analysis. As a sensitivity analysis, we further excluded people who developed dementia up to 3 years after baseline to test the possibility that associations observed were due to reverse causality.

## Results and findings

**Fig. 1:**
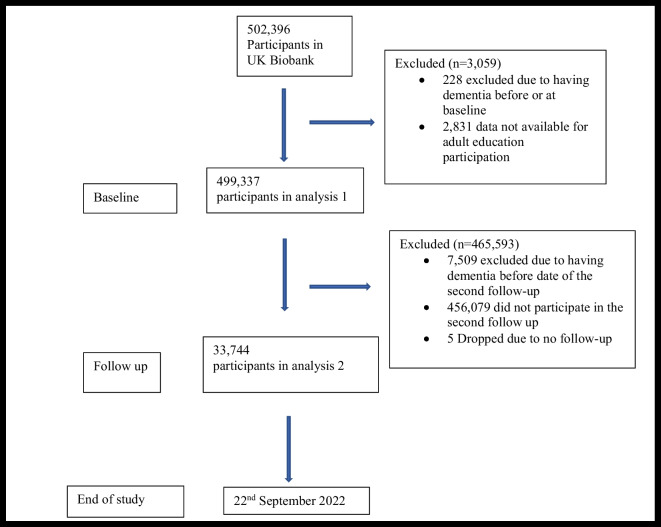
STROBE diagram: participant selection

There were 502,396 potential participants. For analysis 1, we excluded 228 (0.04%) participants as they either had dementia before or at the date of first attended assessment centre at baseline and 2831 (0.56%) participants as they had missing data on adult education participation (see Fig. 1). The mean follow-up time for analysis 1 was 13.2 (standard deviation 2.1) years and a total of 499,337 participants were included in the final sample. For analysis 2, a total of 35,744 people participated in the follow-up assessment. We excluded participants who had dementia before or at the date of the follow-up visit (*N* = 7509) and five participants as they had no follow-up (Fig. 1). The mean follow-up time for analysis 2 was 5.5 (standard deviation 1.3) years.

The mean age at baseline was 56.5 years and 54.4% of the participants were females. There was complete data for age, sex, Townsend deprivation index, excess alcohol consumption, hypertension, diabetes, body mass index and physical inactivity. There was missing data for ethnicity (*N* = 2,674, 0.6%), education (*N* = 2,538, 0.5%), current smoking status (*N* = 1,781, 0.3%) and social contact (*N* = 783, 0.2%). Those who participated in adult education were similar in age to those who did not. Compared to the non-participation group, participants who took part in adult education were more likely to be females. Participants were mostly White (more than 94% in both groups). Townsend deprivation index scores were similar in two groups. Participants who had taken part in adult education class were more likely to be less educated than those who did not participate. They were also more likely to be non-smokers, moderate alcohol drinkers, with no history of hypertension and diabetes, not obese, being more physically active and not socially isolated (see Table 2). For the overall 499,337 participants in baseline sample, 7718 (1.5%) participants developed dementia after baseline, meaning the dementia incidence rate was 15.5/1000 person-years. Seven thousand two hundred thirty-five (94%) of these participants never participated and 483 (6%) participated in adult education (Table 2).

**Table 2:** Baseline characteristics of participants according to baseline participation in adult education

Characteristics	All participants 499,337 (100%)	No participation 463,130 (92.8%)	Participation 36,207 (7.2%)
Age at baseline, years, mean (SD)	56.5 (8.1)	56.4 (8.1)	57.8 (8.0)
Follow-up time, years, mean (SD)	13.2 (2.1)	13.2 (2.1)	13.2 (1.9)
Sex, *n* (%)			
Female	271,724 (54.4)	245,634 (53.0)	26,090 (72.1)
Male	227,613 (45.6)	217,496 (47.0)	10,117 (27.9)
Ethnicity, *n* (%)			
White	471,095 (94.3)	437,124 (94.4)	33,971 (93.8)
Asian	9580 (1.9)	9055 (2.0)	525 (1.5)
Black	7986 (1.6)	7322 (1.6)	664 (1.8)
Chinese	1544 (0.3)	1419 (0.3)	125 (0.3)
Mixed ethnicity	1922 (0.4)	1755 (0.4)	167 (0.5)
Other ethnic groups	4536 (0.9)	4067 (0.9)	469 (1.3)
Unknown	2674 (0.6)	2388 (0.4)	286 (0.8)
Townsend score, mean (SD)	3.0 (1.4)	3.0 (1.4)	3.0 (1.4)
Education, *n* (%)			
Educated beyond age 16	211,206 (42.3)	201,780 (43.6)	9426 (26.0)
Educated ≤ age 16	285,593 (57.2)	259,050 (55.9)	26,543 (73.3)
Unknown	2538 (0.5)	2300 (0.6)	238 (0.7)
Alcohol > 21 units per week, *n* (%)			
Yes	97,239 (19.5)	92,087 (19.9)	5152 (14.2)
No	402,098 (80.5)	371,043 (80.1)	31,055 (85.8)
Current smoking status, *n* (%)			
Current smoker	52,696 (10.6)	50,189 (10.8)	2507 (6.9)
Non-smoker	444,770 (89.1)	411,197 (88.8)	33,573 (92.7)
Unknown	1781 (0.3)	1744 (0.4)	127 (0.4)
Diabetes, *n* (%)			
Yes	25,382 (5.1)	23,905 (5.2)	1477 (4.1)
No	473,955 (94.9)	439,225 (94.8)	34,730 (95.9)
Hypertension, *n* (%)			
Yes	143,798 (28.8)	134,221 (29.0)	9577 (26.4)
No	355,539 (71.2)	328,909 (71.0)	26,630 (73.6)
Body mass index, kg/${\text{m}}^{2}$, *n* (%)			
< 30	375,221 (75.1)	346,785 (74.9)	28,436 (78.5)
≥ 30	124,116 (24.9)	116,345 (25.1)	7771 (21.5)
Physical inactivity, *n* (%)			
Activity levels at or above the WHO’s recommendations	444,971 (89.1)	411,475 (88.9)	33,496 (92.5)
Lower activity levels	54,366 (10.9)	51,655 (11.1)	2711 (7.5)
Social isolation, *n* (%)			
Yes	79,044 (15.8)	71,939 (15.5)	7105 (19.6)
No	419,510 (84.0)	390,456 (84.3)	29,054 (80.2)
Unknown	783 (0.2)	735 (0.2)	48 (0.2)

Only 0.7% of subjects participated in adult education class persistently from baseline assessment to the second follow-up. The majority of participants did not join in adult education class during the study period (see Table 3). After the second follow-up, 209 (0.58%) participants developed dementia in the overall 35,744 participants, giving a dementia incidence rate of 5.8/1000 person-years. Among them, 194 (93%) never participated in adult education, 9 (4%) participated only at baseline, 5 (2%) participated only at the second follow-up and only 1 (1%) consistently participated.

**Table 3:** Change in adult education participation of UK Biobank participants from baseline to follow-up

Adult education class	Frequency	Percentage
Never participated	31,656	88.6
Participated only at baseline	1665	4.7
Participated only at follow-up	2168	6.0
Persistent participation at both baseline and follow-up	255	0.7
Total	35,744	100

### Association of adult education with incident dementia risk

In unadjusted models, and after adjusting for all covariates, we found that participating in adult education class is significantly associated with lower incident dementia risk (hazard ration (HR) = 0.82, *P* < 0.001, 95% confidence interval (CI) 0.74–0.90). The proportionality of hazards assumption was met (see Table 4).

**Table 4:** Association between adult education and incident dementia risk at baseline

Predictor		HR	95% CI	*P* value
Participation in adult education	Model 1	0.85	0.77–0.93	*P* < 0.001
	Model 2	0.74	0.67–0.81	*P* < 0.001
	Model 3	0.80	0.73–0.88	*P* < 0.001
	Model 4	0.83	0.75–0.91	*P* < 0.001
	Model 5	0.82	0.74–0.90	*P* < 0.001

Definition: Model 1 was unadjusted; model 2 was adjusted for age and sex; model 3 was adjusted for education, Townsend score and ethnicity; model 4 was adjusted for hypertension, diabetes, obesity, alcohol, smoking and physical inactivity; model 5 was adjusted for social isolation. Number included in fully adjusted model = 491,143

### Association of change in adult education participation with incident dementia risk

In fully adjusted models, changing or persistent adult education participation was not significantly associated with incident dementia compared to non-participation (Table 5).

**Table 5:** Association between persistent adult education and incident dementia risk

Predictor		HR	95% CI	*P* value
Reference: never participated in adult education	Model 1	1 (1.84) 2 (1.23) 3 (0.51)	0.92–3.67 0.50–3.03 0.07–3.69	1 (*P* = 0.084) 2 (*P* = 0.658) 3 (*P* = 0.508
1- Stopped adult education participation (participated at baseline but not later) 2- Started adult education participation (participated later but not at baseline) 3- Persistent participation	Model 2	1 (1.72) 2 (1.14) 3 (0.34)	0.86–3.44 0.46–2.83 0.05–2.46	1 (*P* = 0.127) 2 (*P* = 0.776) 3 (*P* = 0.286)
	Model 3	1 (1.55) 2 (1.15) 3 (0.34)	0.74–3.23 0.46–2.86 0.05–2.47	1 (*P* = 0.244) 2 (*P* = 0.770) 3 (*P* = 0.287)
	Model 4	1 (1.57) 2 (1.18) 3 (0.35)	0.75–3.29 0.47–2.95 0.05–2.52	1 (*P* = 0.227) 2 (*P* = 0.719) 3 (*P* = 0.296)
	Model 5	1 (1.56) 2 (1.18) 3 (0.35)	0.75–3.27 0.47–2.94 0.05–2.51	1 (*P* = 0.236) 2 (*P* = 0.723) 3 (*P* = 0.293)

Definition: Model 1 was unadjusted; model 2 was adjusted for age and sex; model 3 was adjusted for education, Townsend score and ethnicity; model 4 was adjusted for hypertension, diabetes, obesity, alcohol, smoking and physical inactivity; model 5 was adjusted for social isolation. Number included in fully adjusted model = 30,684

### Sensitivity analysis

Our analyses excluding those who developed dementia within 3 years of baseline assessment gave similar results to our primary analyses (see appendix 1 and 2) whereby participation in adult education at baseline was associated with a lower risk of dementia.

### Brain volume results

There was brain imaging data for 41,695 participants, of whom 38,218 participated in adult education and 3409 did not, with data on adult education participation missing for 68 people. Mean brain volume in those with who did versus did not participate in adult education at baseline was 1.48 mm^3^*10^6^(SD 0.75 mm^3^*10^6^) and 1.49 mm^3^*10^6^(SD 0.74 mm^3^*10^6^) respectively. Total hippocampal volume was similar for both groups as well: 8071 mm^3^ and 7942 mm^3^ respectively. Adult education did not impact on total brain volume (coefficient − 657.4, 95% CI − 2795.1 to 1480.3, *P* = 0.547). Adult education was associated with increased hippocampal volume (coefficient 33.9, 95% CI 8.9 to 59.0, *P* = 0.008). Both analyses were adjusted for age, sex, education, deprivation, ethnicity, hypertension, diabetes, ethnicity obesity, smoking, alcohol use, physical inactivity and social isolation.

## Discussion

In this large longitudinal cohort study of UK adults, we examined the association of adult education and dementia risk and examined potential mechanisms for the association. We replicated the finding that participation in adult education is associated with a reduced risk of developing incident dementia. However, there was no convincing evidence that changing or persistent participation in adult education class was associated with incident dementia risk. Analysis of brain volume indicated that adult education may have a protective effect by increasing hippocampal size or slowing volume loss, in line with the cognitive reserve hypothesis.

The finding that adult education was associated with incident dementia risk is consistent with previous studies on the association of cognitively stimulating activities and incident dementia prevalence [22]. The positive effect of adult education on reducing incident dementia risk also corresponds with the view that cognitive leisure activities can be beneficial on cognition and can protect against dementia [23]. Our study builds this evidence base through its long duration of follow-up. A previous systematic review of the effect of cognitive stimulating activities on incident dementia mostly included studies with less than 6 years interval between activity assessment and dementia ascertainment [7]. This current UK Biobank study improved on this by having a mean follow-up time of 13.2 years from baseline and found similar results. As symptoms of dementia can emerge up to 12 years prior to dementia onset, links between a putative risk factor and dementia onset with a shorter interval than this may be due to the factor developing as an early symptom or consequence of dementia, rather than it acting as a risk factor. Our result is less likely to be due to reverse causation and suggests more strongly that adult education might lower the risk of developing incident dementia.

There are several potential mechanisms by which adult education could reduce the risk of dementia. Firstly, cognitive reserve capacity could explain this positive result. Evidence had shown that lifelong education could significantly lower the risk of developing dementia [24] by building cognitive reserve, and it is plausible that this can be further expanded by ongoing involvement in adult education. Adult education may also act through other mechanisms, such as providing social contact with others [6, 25], encouraging healthier lifestyle choices like drinking less alcohol or taking exercise, or reducing stress.

Our finding that hippocampal volume was greater in those who participated in adult education suggests a potential mechanism for the association we found between education and dementia risk. Other studies have found that education in early life is associated with less age-related reductions in hippocampal volume [26, 27]. This is taken, by the cognitive reserve hypothesis, to indicate that the cognitive effort associated with education builds greater brain substrate and this may reduce susceptibility to neuropathology and/or its effects on cognition and functioning. Our study suggests, to our knowledge, for the first time, that this potential mechanism may also apply to participation in adult education.

Though the results of our analysis of the association of dementia with changing adult education participation were not statistically significant, the numbers in these analyses were small and persistent adult education participation was in the direction of being associated with decreased incident dementia risk as we hypothesised. In the Whitehall social activities study, decline in participation in leisure activities over 10 years was associated with an increased risk of subsequent incident dementia [28]. This study found results consistent with this. Compared with persistent adult education participation, participants who only took part in baseline and the follow-up both showed higher dementia risks (not statistically significant) which may partly reflect this group developing cognitive decline, resulting in reducing participation in activities, i.e. reverse causality [29].

### Strengths and limitations of the study

This study has several strengths including (1) its large sample size, (2) the follow-up times were relatively long compared to previous studies so that participant’s dementia outcomes could be tracked after baseline assessment and the long-term positive effect of adult education could also be examined and (3) a wide range of confounders were controlled for in the analyses.

However, some limitations still need to be addressed. Firstly, there may be inaccuracies in recording of dementia as this was based on self-report or linked health records. People developing dementia might not seek treatment in health services and, as a result, some missing dementia cases might not be reported and recorded in the electronic health records from where we ascertained dementia cases. This may vary by socio-demographic characteristics, such as dementia being less likely to be recorded in people from minority ethnic groups [30], resulting in ascertainment bias. Secondly, the sample size for the second analysis was too small as the second assessment of adult education participation was only conducted in a subsample. However, the findings in this analysis, though underpowered, were consistent with the main study results and there was limited missing data in the study overall. Additionally, we had no information about type of adult education which was engaged in. This would have been helpful to ascertain if particular types of education are more helpful than others.

A further limitation to be considered is that the UK Biobank participants may not be fully representative of the general population. In the recruited cohort, participants tend to live in less socioeconomically deprived areas. They are less likely to be obese, to smoke and to drink excess alcohol on a daily basis. They also have reported fewer health-related conditions, which means they are healthier than the general population [31]. These characteristics, as well as the limited diversity in terms of ethnicity, means that caution is needed about the generalisability of these groups and that the research should be replicated in other populations.

What also needs to be considered is that the positive effect of participation in adult education class might be confounded by effect of other leisure activities, for example physical activities. An Italian cohort study had shown that physical activities reduced risk of vascular dementia [32] and that this was related to higher hippocampal and total brain volumes [33]. Similarly, social engagement was found to be beneficial for reducing overall dementia risk in ageing population as well [34]. We tried to account for this by adjusting for social contact and physical activity, but it is not possible to rule out residual confounding.

## Conclusions

In conclusion, participating in adult education class was associated with a lower risk of incident dementia but persistent or changing participation in adult education did not show a statistically significant association with incident dementia risk. We found that adult education may confer protective effect against dementia by increasing hippocampal volume. Future study should consider interventional studies to compare the effect of increased adult education on improving overall cognition in older adults. Furthermore, considering the pressing need to support older adults to reduce dementia risk, and the potential general benefits for health of education, policy makers should consider how adult education could be implemented on a wider scale.

## Supplementary Information

Below is the link to the electronic supplementary material.

Supplementary file1 (DOCX 20 KB)
