# Supplementary material for: Association between adult education, brain volume and dementia risk: longitudinal cohort study of UK Biobank participants
Source: GeroScience. 2024 Jul 19;47(1):903–13. doi: 10.1007/s11357-024-01285-y (PMC11872847; doi:10.1007/s11357-024-01285-y)
Supplement: Supplementary file 1 — Supplementary file1 (DOCX 20 KB) [file 11357_2024_1285_MOESM1_ESM.docx]

**Appendices**

**Appendix 1: Association between adult education and incident dementia risk at baseline after excluding study participants who developed dementia within 3 years of adult education assessment**

| **Predictor** |  | HR | 95% CI | P value |
| --- | --- | --- | --- | --- |
| **Participation in adult education** | Model 1 | 0.84 | 0.76-0.92 | P<0.001 |
|  | Model 2 | 0.81 | 0.73-0.89 | P<0.001 |

**Definition:** Model 1 was unadjusted, model 2 was adjusted for age, sex, education, Townsend score, ethnicity, hypertension, diabetes, obesity, alcohol, smoking, physical inactivity and social isolation. Number included in fully adjusted model=490,886. Total of 267 incident dementia cases.

**Appendix 2: Association between persistent adult education and incident dementia risk after excluding study participants who developed dementia within 3 years of adult education assessment**

| **Predictor** |  | HR | 95% CI | P value |  |  |
| --- | --- | --- | --- | --- | --- | --- |
| **Reference: never participated in adult education** | Model 1 | 1 (1.84)  2 (1.23)  3 (0.51) | 0.92-3.67  0.50-3.03  0.07-3.69 | 1 (P=0.084)  2 (P=0.658)  3 (P=0.508) |  |  |
| 1. **Stopped adult education participation** 2. **Started adult education participation** 3. **persistent participation** | | Model 2 | 1 (1.56)  2 (1.18)  3 (0.35) | 0.75-3.27  0.47-2.94  0.05-2.51 | 1 (P=0.236)  2 (P=0.723)  3 (P=0.293) | |

**Definition:** Model 1 was unadjusted, model 2 was adjusted for age, sex, education, Townsend score, ethnicity, hypertension, diabetes, obesity, alcohol, smoking, physical inactivity and social isolation. Number included in fully adjusted model=30,684. Total of 267 incident dementia cases.
